# Sleep and Psychosocial Risk Factors Associated with Social Jet Lag and Sleep Duration Among Colombian University Students

**DOI:** 10.3390/clockssleep7040064

**Published:** 2025-11-07

**Authors:** Andrés Camargo, Leandro P. Casiraghi, Diego A. Golombek, Edith Villalobos, Viviana González, Carlos Orozco, Elena Jiménez, Danny Sanjuanelo, Oscar Pianeta, Rafael Vargas

**Affiliations:** 1 School of Health and Sport Sciences, Fundación Universitaria del Área Andina, Bogotá 111511, Colombia; 2 Laboratorio Interdisciplinario del Tiempo (LITERA), Universidad de San Andrés—CONICET, Buenos Aires 1644, Argentina; lcasiraghi@udesa.edu.ar (L.P.C.); dgolombek@udesa.edu.ar (D.A.G.); 3 Facultad de Administración, Finanzas y Ciencias Económicas, Vicerrectoría de Innovación Académica, Universidad Ean, Bogotá 110231, Colombia; gevillalobos@universidadean.edu.co; 4 School of Nursing, Fundación Universitaria del Área Andina, Bogotá 111511, Colombia; vigonzalez2@areandina.edu.co; 5 Instituto Nacional de Cancerología, Bogotá 111511, Colombia; corozco@cancer.gov.co; 6 School of Nursing, Universidad Mariana, Pasto 520001, Colombia; mejimenez@umariana.edu.co; 7 Departamento de Ciencias Exactas y Naturales, Universidad de Ciencias Aplicadas y Ambientales (U.D.C.A), Bogotá 111166, Colombia; dsanjuanelo@udca.edu.co; 8 School of Medicine, Universidad de Ciencias Aplicadas y Ambientales (U.D.C.A), Bogotá 111166, Colombia; opianeta@udca.edu.co; 9 Facultad de Medicina y Ciencias de la Salud, Universidad Militar Nueva Granada (UMNG), Bogotá 110111, Colombia

**Keywords:** social jet lag, sleep deprivation, university students, chronotype, circadian rhythm

## Abstract

Undergraduate students and healthcare professionals often experience irregular sleep patterns, social jet lag (SJL), and rotating shifts that affect their performance. This study examined the association between SJL, sleep duration, and psychosocial factors among 1409 Colombian undergraduate students (mean age 24.4 ± 6.7 years) using data from the Ultra-Short Version of the Munich ChronoType Questionnaire collected between June and September 2023. Multivariable linear regression analysis identified factors associated with SJL. The prevalence of SJL exceeding two hours was high (84.6%), with an average magnitude of 4.4 h. Chronotype (MSFsc) was negatively correlated with SJL, indicating that students with later chronotypes tended to experience greater misalignment between biological and social time. Younger age and a higher number of working days were significantly associated with increased SJL, whereas substance use and mental health history showed no significant effects. These findings highlight that work-related demands, particularly frequent working days, play a key role in exacerbating social jet lag. The results underscore the need for institutional strategies to promote sleep health among Colombian university students and health professionals.

## 1. Introduction

Biological rhythms, including the 24-h circadian rhythm, regulate essential functions, ranging from metabolic processes to behavioral and molecular activities. These rhythms vary in cycles ranging from minutes to months and years. Circadian rhythms are synchronized with external cycles, also known as zeitgebers [1]. Zeitgebers are environmental cues, such as light, that help regulate the biological clock and facilitate the synchronization of circadian rhythms with the 24-h daily cycle [2].

Chronotypes refer to the natural variation in individual preferences and tendencies for sleep and wake times, broadly categorized into “morning types” (or larks), “evening types” (or owls), and “intermediate types.” Morning types tend to wake up early, peak in alertness and productivity during the first part of the day, and prefer to go to bed early. Evening types, on the other hand, naturally wake up later, reach peak performance in the late afternoon or evening, and prefer to stay up late. Intermediate types fall between these two extremes, exhibiting flexibility in their sleep and wake patterns that allows them to adapt more easily to the standard societal schedule [3,4]. These chronotypes are influenced by genetic, environmental, and age-related factors, with adolescents and young adults more likely to exhibit evening-type preferences. Other zeitgebers, such as meals, exercise, and social interactions, also play a role in regulating circadian rhythms [2].

In addition, the social clock serves as the reference for coordinating activities in a specific region or time zone [5]. It is essential for ensuring punctuality in daily activities such as attending school, working, or using public transportation. These social activities, including regular commuting to school or work, also act as zeitgebers, providing consistent signals that help regulate circadian rhythms [6].

Technological advancements have led to an increase in indoor time, often away from natural light, and a greater reliance on artificial lighting even at night. This decrease in zeitgeber exposure has had significant consequences [7]. For most people, there has been an increase in the variability of chronotypes, defined as the time of day with increased activity, and a shift toward later, or evening, hours. It is important to note that while our internal biological clocks have undergone significant changes, the social clock, which reflects social schedules, has not kept pace. This has resulted in an increasing misalignment between the two temporal references, commonly referred to as ‘social jet lag’ [5,7].

Social jet lag (SJL) is a prevalent phenomenon in the workforce of industrialized nations, affecting a significant proportion of students and employees [4,8]. This phenomenon is often linked to sleep deficits, although it is still uncertain whether its effects are primarily due to circadian misalignment or sleep deprivation [9]. Fixed schedules for school or work are common, and may not align with individuals’ circadian preferences. As a result, individuals tend to follow activity patterns that align with their natural circadian rhythms during their days off [9,10,11].

SJL can have negative health consequences, including cognitive impairment, reduced alertness, sleep disorders, mood disturbances, and an increased risk of metabolic diseases such as obesity and diabetes [12,13]. Additionally, its potential relationship with psychiatric disorders, such as depression, anxiety, stimulant use, and ADHD, has been explored, although results are mixed among studies [14,15,16,17,18].

Despite the growing body of research on circadian rhythms and SJL, much of the existing knowledge comes from high-income countries. There is a significant need to investigate how these concepts affect different populations, particularly in low- and middle-income contexts such as Colombia. Moreover, while the impact of SJL on physical and mental health has been extensively documented, few studies have examined its relationship with substance use and psychological disorders. Substance abuse has been suggested to have a bidirectional relationship with sleep disorders, but the connection between circadian misalignment and substance dependence remains uncertain. Recent research has shown that SJL can affect psychological well-being and contribute to behavioral issues in young adults, especially in different sociocultural contexts [19].

The present study aimed to examine the associations between SJL, sleep duration, and chronotype among Colombian university students. The primary aim was to determine the relationship between chronotype, as defined by Roenneberg et al. (2003) [20] and adapted by Ghotbi et al. (2020) [21] as the midpoint of sleep, on free days corrected for sleep debt (MSFsc). The secondary aim was to evaluate the influence of psychosocial and behavioral factors—such as socioeconomic status, work schedule, substance use, and mental health history—on the magnitude and duration of SJL.

## 2. Results

A total of 1409 university students participated (mean age = 24.4 years, SD = 6.7). The distribution by gender included 398 men (28.3%) and 1011 women (71.7%). Most participants were classified within low-to-middle socioeconomic strata (SES 1–3), as shown in Table 1.

The chronotypes were distributed into five quintiles (Q1–Q5) based on the mid-sleep time corrected for sleep debt (MSFsc), categorized as follows: Q1 (early morning chronotype), Q2 (early intermediate), Q3 (late intermediate), Q4 (late), and Q5 (extremely late chronotype). The distribution of chronotypes based on the mid-sleep time (MSFsc) is shown in Figure S1 (Q5, which included only one participant, was excluded from the main analysis and is provided as Supplementary Materials).

Lifetime history of psychiatric diagnoses indicated that anxiety (7.5%) and depression (1.3%) were the most frequently reported conditions. A Chi-square test was applied to assess the differences between men and women, revealing that men reported higher percentages of both anxiety and depression compared to women (*p* < 0.01). In terms of current psychiatric medication, 8.8% of participants reported being under active treatment (Table 1).

Substance use was prevalent, with caffeine being the most commonly used substance, followed by alcohol and tobacco. Notable gender differences were observed in substance use, with men reporting higher percentages of alcohol and tobacco use, while women had higher percentages of caffeine and vaper use (Table 1).

In our study, it was found that 46% of students sleep less than 6 h per day during the week, while 75.2% sleep more than 6 h on non-workdays. The average weekly sleep duration was 5.4 h (SD= 3.0 h), increasing to 7.6 h (SD = 2.3 h) on non-workdays. The prevalence of SJL within the range of 1 to 2 h was 15.4%, with 84.6% experiencing SJL exceeding 2 h. The participants, on average, had a social jet lag of 4.4 h (SD = 3.4 h; range = 0–11 h; Table 2).

On the other hand, a similar proportion of participants from both genders reported having worked rotating shifts in the past three months. However, significant disparities emerged in work shift distribution, with a higher percentage of men engaged in night shifts, while more women worked during the day (*p* < 0.01) (Table 3).

In terms of working hours, men were more likely to work fewer hours per week (up to 36 h), while women were more likely to work moderately more hours (37 to 48 h; *p* < 0.05). Sleep duration on workdays and non-workdays did not exhibit significant gender differences, suggesting that both men and women maintained similar sleep patterns (Table 3).

Table 4 presents Spearman’s correlations highlighting statistically significant associations between key variables. Firstly, SJL demonstrated a positive correlation with the number of working days (r = 0.076, *p* < 0.001), suggesting that as individuals engage in more workdays, their SJL tends to increase. Conversely, SJL displayed a negative correlation with the number of free days (r = −0.076, *p* < 0.001), indicating that individuals with more free days may experience reduced SJL. Additionally, SJL demonstrated a strong and significant positive correlation with sleep duration on non-workdays (r = 0.583, *p* < 0.01), indicating that individuals with greater SJL tend to have longer sleep duration on non-workdays. Conversely, there was also a substantial but negative correlation between SJL and sleep duration on workdays (r = −0.736, *p* < 0.001), suggesting that higher SJL is associated with shorter sleep duration on workdays (Table 3).

Regarding chronotype, we observed a significant negative correlation with SJL (r = −0.311, *p* < 0.001). This suggests that as SJL increases, individuals with later chronotypes (higher MSFsc) tend to experience greater misalignment between their biological and social time. In addition, chronotype was strongly correlated with sleep duration on workdays (r = 0.839, *p* < 0.001) and on non-workdays (r = 0.523, *p* < 0.001). This suggests that individuals with a later chronotype tend to have longer sleep durations on both workdays and free days (Table 3).

Furthermore, sleep duration on non-workdays exhibited a negative correlation with age (r = −0.081, *p* < 0.001), suggesting that as individuals age, their non-workday sleep duration may decrease. Additionally, sleep duration on non-workdays displayed a negative correlation with sleep duration on workdays (r = −0.127, *p* < 0.01), indicating that individuals with longer workday sleep tend to have shorter non-workday sleep (Table 4).

The multiple linear regression analysis revealed a significant model fit (R^2^ = 0.159). Individuals with an early chronotype tend to experience more SJL (β 95% CI ranged from −0.025 to −0.018). Age was negatively associated with SJL, implying that as younger the individual, their SJL tends to increase (β 95% CI: −4.942 to −1.618). In the same direction, a higher number of working days during a typical week was linked to increased SJL (β 95% CI: 2.340 to 10.583., suggesting that work-related factors can disrupt sleep alignment. Furthermore, the number of working days during a usual week revealed a positive association with SJL, suggesting that a higher number of working days during a typical week is associated with increased SJL (β 95% CI: 2.340 to 10.583) (Table 5).

The analysis of variance (ANOVA) revealed significant differences in SJL among groups defined by the consumption of various substances (Table 6). However, the Bonferroni post hoc tests did not confirm these differences, as no significant differences in SJL were found between the groups defined by the consumption of any substances, including alcohol, cigarettes, coffee, vaping, or a combination of these. The Bonferroni correction did not support the initial findings, and no significant differences were observed after adjustment.

Similarly, regarding the differences in SJL among groups based on personal history of mental health issues, the ANOVA results showed a statistically significant difference in SJL among these groups (*p* < 0.05). However, the Bonferroni post hoc comparisons revealed no statistically significant differences in SJL between any of the groups defined by their personal history of mental health issues. This discrepancy suggests that while global differences in SJL were observed, the specific pairwise comparisons did not confirm these findings. This could be due to variability within the groups or limitations in statistical power during the post hoc tests. Therefore, these results should be interpreted with caution.

## 3. Discussion

Our data show that in a sample of 1409 health sciences university students, sleep patterns exhibited a noteworthy prevalence of insufficient sleep during the week (46%), emphasizing a potential public health concern [10,22]. A lifetime psychiatric history revealed a significant prevalence of anxiety and depression, with men reporting higher percentages than women. Moreover, our findings highlighted a strong association between chronotype and social jetlag, with participants exhibiting significant circadian misalignment, particularly those with later chronotypes. Current psychiatric medication usage was reported by 8.8% of participants, while substance use, primarily caffeine, was also prevalent. Interestingly, some studies have suggested that SJL can encourage certain habits, including smoking, as a way to manage its effects [23]. However, no significant relationship was found between substance use and SJL in our study.

SJL, which reflects the misalignment between social and biological time, was observed in a significant portion of the participants, with an average duration of 4.4 h. The relationship between SJL and sleep timing showed that individuals with greater SJL tended to have earlier sleep midpoints compared to those with lower levels of SJL. This finding is consistent with studies in both questionnaire-based and laboratory-based settings, including those with college student samples, which have consistently reported a high prevalence of shorter sleep on workdays compared to days off and elevated SJL [24,25]. In health sciences faculties, extended shifts and brief sleep periods are the norm. This pattern is closely linked to heightened mental and physical strain, contributing to mental health issues [26]. Other studies have indicated that working hours are associated with a decline in mental health, even after adjusting for potential biases from unobservable individual factors. This pattern is similarly observed in real-world conditions, where external factors such as shift work and academic demands might lead to greater SJL, compared to more controlled laboratory settings where these factors are minimized, suggesting that prolonged working hours may increase the risk of developing depressive disorders [17,27]. While similar patterns have been observed in Western countries, where students balance academic and work demands, their longer work hours, driven by higher living costs, may exacerbate SJL. Further research is needed to explore the impact of work hours on SJL in Colombian students, considering how cultural, socioeconomic, and academic factors may influence their sleep patterns.

Furthermore, our findings are consistent with previous studies reporting substantial variability and meaningful levels of SJL among university students. In undergraduates, higher SJL has been linked to poorer weekly academic performance during terms with fixed schedules [28]. This comparison highlights how in real-life conditions, where students experience varying workloads and irregular schedules, sleep patterns and jet lag impact academic outcomes, unlike controlled laboratory conditions where external factors are more stable. Likewise, a difference greater than two hours between the mid-sleep point on workdays and free days has been treated as a behaviorally and physiologically relevant circadian misalignment [29].

It is important to highlight that, according to studies, the socio-economic background of women, particularly those in lower SES groups, can impact their sleep patterns and overall well-being. Studies suggest that socio-economic factors, including lower SES, can contribute to greater sleep disturbances, higher levels of sleep inertia, and reduced sleep quality, particularly among women [30]. This socio-economic factor should be considered in interpreting the sleep patterns and SJL observed in this study.

This misalignment is more pronounced in self-reported data, where participants provide their sleep timing in real-world conditions as opposed to controlled laboratory environments, where sleep timing is generally more regulated. This inconsistency in sleep timing on both workdays and free days suggests that social jetlag (SJL) results from a misalignment between an individual’s biological rhythms and societal schedules. Our study offers new insights into this phenomenon, particularly in the context of Colombian students, where socioeconomic and academic factors may exacerbate the misalignment between biological and societal time compared to students in Western countries. This misalignment, particularly pronounced among individuals with later chronotypes (those with higher MSFsc values), aligns with findings from other studies suggesting greater difficulty aligning sleep patterns with societal demands, further exacerbating SJL [14,31]. However, while similar studies in Western countries have indicated that evening types exhibit more SJL than morning types, our study highlights the importance of contextual factors such as cultural and socioeconomic conditions, which may have an additional influence on the severity of SJL in Colombian students. Interestingly, earlier research has presented contrasting findings, indicating that evening types exhibit more SJL than morning types [14,31]. Additionally, it has been reported that morning types tend to maintain greater regularity than evening types among both adolescents and adults [32,33]. Overall, our findings confirm previous observations from laboratory and Western-based studies regarding the association between chronotype and SJL but also provide novel evidence from a Latin American population. This suggests that the mechanisms underlying SJL are context-dependent rather than universal, influenced by sociocultural and economic factors unique to this region. By highlighting these contextual nuances, our study extends current knowledge on circadian misalignment and underscores the need for culturally adapted strategies to mitigate its effects. These contrasting findings illustrate the complexity of the relationship between sleep patterns, social disengagement, and mental health in adolescents, suggesting the need for personalized interventions that take into account individual chronotypes and real-life schedules.

Regarding chronotypes, we divided participants into quintiles based on their MSFsc values, where Q1 represented the earliest chronotypes and Q5 represented the latest. This division allowed us to categorize individuals based on the midpoint of their sleep during free days, corrected for sleep debt, into five distinct groups. This continuous measure enabled a more precise identification of chronotypes. As the chronotype moved from earlier (Q1) to later (Q5), the participants reported greater SJL, further emphasizing how this misalignment between biological and societal time is more pronounced when individuals’ sleep patterns are more detached from their natural circadian rhythms.

The significant negative correlation between chronotype and SJL, further confirmed in the multiple regression analysis, could be partially attributed to the geographical location of Colombia and light exposure. Situated near the equator, Colombia experiences relatively consistent day lengths throughout the year, which correlates with an early chronotype on average, in contrast to geographical locations further west, where a later chronotype is more common [34,35,36].

In examining the interplay between sleep inertia and SJL, we found that individuals with evening chronotypes are more vulnerable to sleep inertia, which reduces cognitive function and alertness immediately upon waking. Previous studies suggest that evening types experience greater sleep inertia due to SJL, particularly in real-world conditions where social obligations force them to adjust their sleep patterns against their natural circadian rhythms. This misalignment between biological and social clocks contributes to greater difficulty transitioning from sleep to wakefulness, exacerbating sleep inertia and daytime dysfunction. Our results reflect these findings, showing that evening types report greater sleep inertia, which likely contributes to their poorer sleep quality and higher daytime dysfunction [37,38].

The results of the multiple linear regression analysis indicated several factors associated with SJL. Individuals with a later chronotype experienced less SJL, potentially due to their natural inclination to align their sleep patterns with their daily routines more effectively. Age was negatively associated with SJL, implying that as individuals grow older, their SJL tends to decrease. This finding has been reported in a previous study with a large sample showing that SJL decreases across the lifespan [37]. However, this association should be interpreted with caution, as our sample consisted mainly of young adults, reflecting the typical age range of university students. Therefore, the variability observed likely represents minor intra-group differences rather than developmental changes across the lifespan. Age was included as a covariate to control for small within-group variations rather than to infer age-related circadian effects.

It is important to note that approximately half of our participants reported having worked rotating shifts in the past three months, which could influence their sleep patterns and the experience of SJL. This shift work may confound the findings, particularly those related to sleep deprivation and SJL. Therefore, the interpretation of sleep and SJL patterns should be made with caution, considering the potential confounding effect of shift work.

Future research should include diverse academic and non-academic samples to determine whether these patterns are consistent across broader populations. In addition, the use of the corrected formula for social jet lag (SJLsc), which excludes the effects of sleep debt, could provide a more accurate representation of the misalignment between biological and social time. This adjustment would allow for a more precise understanding of circadian misalignment and its physiological consequences, offering valuable insights into how SJL affects health and well-being. Such methodological improvements could contribute to a deeper exploration of the relationship between SJL and various health outcomes, ultimately supporting the development of more effective interventions. These considerations should be taken into account when interpreting the present findings, as discussed in the study limitations.

### Limitations

One limitation of our study is the use of the μMCTQ to assess chronotype and SJL. Although this questionnaire is extensively validated and suitable for a wide range of work schedules, approximately half of our participants reported having worked rotating shifts in the past three months. It is important to note that, although their primary activity was studying and not all participants worked every night, the μMCTQ may not be the most accurate tool for this particular group. The MCTQShift, specifically developed for shift workers, could offer greater accuracy in assessing chronotype in these cases. However, highly irregular shift schedules, such as those commonly encountered in hospitals, may still present challenges in chronotype assessment, even with the MCTQShift [39].

Another limitation of our study is the reliance on self-reported data to assess sleep patterns and chronotype, without the inclusion of objective measures such as actigraphy. While self-reports are widely used in sleep research, they are subject to biases, such as recall bias or social desirability bias, which can affect the accuracy of the data. Future studies should aim to incorporate objective tools such as actigraphy or polysomnography to provide more precise and reliable measurements of sleep duration and quality. This would allow for a more accurate assessment of sleep patterns and their relationship with SJL and other health outcomes.

Another limitation of our study is the gender imbalance in the sample. The sample is heavily skewed towards females, which may affect the generalizability of the results. We acknowledge that this bias could potentially influence the findings and their applicability to the broader population. Furthermore, recent studies have reported higher sleep irregularity, greater sleep inertia, and role overload among female students and healthcare trainees compared with males [36,40]. Female students may also face sociocultural pressures for earlier wake times, which can disrupt sleep timing and exacerbate misalignment between circadian and social clocks, contributing to more pronounced sleep disturbances and reduced daytime performance [36]. These findings align with our results, given the predominance of female participants in the sample, which may partly explain the observed variability in sleep patterns and SJL.

It is important to highlight that there are fundamental differences in circadian rhythms between males and females that may contribute to the observed SJL. Research has shown that women tend to have a greater tendency towards ‘eveningness’ and are more susceptible to the negative effects of SJL, such as decreased academic performance and sleep quality. These differences may be related to biological and social factors that affect circadian rhythms and their alignment with social demands [40].

Additionally, the cross-sectional design of this study limits the ability to establish causal relationships between SJL, sleep inertia, and mental health-related variables. Therefore, the observed associations should be interpreted as correlational rather than causal. Future longitudinal studies are necessary to determine the directionality and temporal dynamics of these associations.

Moreover, because the study focused exclusively on health sciences university students, the findings should not be generalized to other age groups or adolescent populations. One limitation of this study is the geographical focus on university students from Bogotá and Pasto. While these cities share similar photoperiods and academic/work schedules, they differ in urbanization and socioeconomic conditions, which may influence sleep timing and SJL. Therefore, caution should be exercised when generalizing these findings to other regions or countries with different environmental and social contexts.

While the ANOVA indicated significant differences in SJL between the substance use and mental health history groups, the post hoc tests failed to confirm these differences. This discrepancy suggests that, although a global difference was detected, specific group comparisons did not yield consistent results. This could be due to the variability within groups or limitations in statistical power when conducting pairwise comparisons, highlighting the need for further investigation with larger and more homogeneous groups.

The regression model (R^2^ = 0.159) indicates a modest explanatory power. While this is acceptable in behavioral research, it suggests that additional variables not considered in the current study might be influencing SJL. Future studies should aim to explore these variables to enhance the explanatory power of the model and deepen our understanding of the complex factors contributing to SJL.

## 4. Materials and Methods

### 4.1. Participants

University students enrolled in private and public universities in Colombia from two cities, Bogotá (4.710989° N, 74.072092° W) and Pasto (1.2136° N, 77.2811° W), were invited to participate through institutional mail. We created and compiled all questionnaires using a Google Forms internet-based survey. Their work or school schedules range from 7:00 to 20:00, with an average class time varying between 6 and 8 h, considering attending courses as classified on a “workday”.

Data collection was carried out between June 2023, when sunrise was at 05:42 and sunset at 18:05, and continuing until September 2023, when sunrise was at 05:43 and sunset at 17:47. Colombia is a country with constant photoperiods; sunrise and sunset times in Colombia do not significantly differ throughout the year. Students over 18 years old of both genders were included. Regular classes were in session during this period.

The study was approved by the University Foundation of the Andean Area Health Sciences Institutional Review Board. After completing informed consent, participants were asked to complete the internet-based survey.

### 4.2. Measures

#### 4.2.1. Sleep Variables

The μMCTQ (Ultra-Short Version of the Munich ChronoType Questionnaire) was used to assess chronotype, defined as the mid-sleep point on free days corrected for sleep debt (MSFsc), and to estimate sleep duration on workdays and non-workdays [21]. It calculates parameters like midpoint of sleep during days off corrected for sleep debt on workdays (MSFsc), mid-sleep point on free days (MSF), and mid-sleep on workdays (MSW) based on sleep onset (SO) and sleep end (SE) [33]. The midpoint of sleep falls between SO and SE [31]. MSF is the chronotype marker when workday sleep duration is greater than or equal to free day sleep duration, while MSFsc is the core marker when free day sleep duration surpasses workday sleep duration [21].

Sleep duration on workdays and non-workday was computed using the formulas outlined in Ghotbi et al. (2020) [21]. The calculation for sleep duration on work-free days involved subtracting the sleep onset (SOf) from the sleep end (SEf), while the sleep duration on workdays was determined by subtracting the sleep onset on workdays (SOw) from the sleep end on workdays (SEw). Subsequently, these measures were converted into minutes by multiplying the values by 24 and 60.

Chronotype (MSFsc) was computed following the methodology reported by Ghotbi et al. (2020) [21] this correction is a linear one, considering the weighted average of sleep duration across the entire week (SDweek) and the sleep duration on work-free days (SDf). The difference between these two values is used to estimate how much longer individuals sleep on a work-free day compared to a scenario where they have no prior sleep debt. If SDf is less than or equal to SDw, the midpoint of sleep corrected for sleep debt (MSFsc) remains equal to MSF. However, if SDf is greater than SDw, MSFsc is calculated as MSF—(SDf-SDweek)/2 [21].

While the μMCTQ is widely used to assess chronotype and SJL, it has limitations when applied to shift workers. Specifically, shift workers exhibit greater variability in sleep–wake cycles, and the μMCTQ may not accurately capture the sleep patterns of individuals with irregular work schedules.

Chronotypes were divided into quintiles based on MSFsc values, with Q1 representing the earliest chronotypes and Q5 representing the latest. This continuous measure allowed us to identify chronotypes more precisely, with SJL increasing as individuals moved from earlier to later chronotypes.

SJL was determined by computing the absolute difference between the mid-sleep point on workdays (MSW) and the mid-sleep point on free days corrected for sleep debt (MSFsc). This variable was treated as a continuous measure, where greater differences indicated higher levels of misalignment between biological and social time. SJL greater than 2 h was considered clinically relevant circadian misalignment, a threshold widely used in chronobiological research to distinguish individuals with meaningful social–biological desynchrony from those with minimal misalignment [27].

In this study, chronotype refers specifically to the midpoint of sleep on free days corrected for sleep debt (MSFsc), as previously defined by Roenneberg et al. (2003, 2012) [20,29]. This parameter was used instead of categorical classifications (e.g., ‘morningness’ or ‘eveningness’) to provide a continuous and objective estimate of individual circadian phase.

#### 4.2.2. Sociodemographic and Mental Health Variables

A self-administered questionnaire was used to collect sociodemographic and psychosocial information, including age, gender, socioeconomic stratum (SES), and mental health history. In Colombia, SES classification is determined by the National Administrative Department of Statistics (DANE) based on criteria such as land use, access to public utilities, road accessibility, terrain, property value, and housing characteristics. SES 1 represents the lowest socioeconomic level, while SES 6 corresponds to the highest.

Participants also reported their lifetime history of psychiatric diagnoses confirmed by a mental health professional (e.g., anxiety, depression, ADHD, or sleep disorders), current use of psychiatric medication, and current substance use (alcohol, tobacco, caffeine, vaping, or mixed).

In addition, information regarding employment conditions was collected, including experiences with rotating shifts during the previous three months, working on non-workdays, the number of working days per week, and the number of days off. These variables were included to evaluate the potential influence of occupational schedules on sleep patterns and SJL.

#### 4.2.3. Employment Status and Work Schedule

Employment status referred to paid technical or service-related work performed outside the university in addition to academic activities. In Colombia, many undergraduate students, particularly those enrolled in health sciences programs, work part-time or full-time in healthcare-related technical positions—most commonly as nursing assistants or in patient-care support roles—to finance their studies and living expenses. These work activities often involve irregular or rotating schedules, including night shifts. Therefore, information regarding the type of work schedule (day, night, or rotating shifts) and the average number of weekly working hours was collected to evaluate its potential influence on sleep duration and SJL.

### 4.3. Data Analysis

We assessed the normal distribution of variables examining the skewness and kurtosis, following the established criteria for large sample sizes (where >300) [41].

The analysis began with descriptive statistics for the variables. Spearman’s correlation coefficient was utilized to examine the relationships between continuous variables and SJL.

Multiple linear regression using the stepwise forward method was carried out, with SJL as the dependent variable, after checking multicollinearity, and confirming the homoscedasticity of residuals. The model provided the coefficient of determination (R2), unstandardized regression estimate (B), and the respective standard error (SE) along with 95% confidence intervals (95% CI). The final model retained was the one with the highest R2 and included only variables that displayed a statistically significant association (*p* < 0.05).

To further evaluate the variance differences for SJL based on lifetime history of psychiatric diagnoses and current substance use categories, one way ANOVA with post hoc comparisons using Bonferroni correction was applied. Statistical significance was set at *p* < 0.05.

Data analysis was performed using SPSS v. 29.

## Figures and Tables

**Table 1:** Sociodemographic and Mental Health Factors in the included sample.

	Men (n = 398)	Women (n = 1011)	Total Sample (N = 1409)
Age, Mean (SD)	25.9 (8.1)	23.9 (6.0)	24.4 (6.7)
Socioeconomic Stratum (SES), n (%) *			
1	190 (47.7)	566 (56.0)	756 (53.7)
2	199 (50.0)	434 (42.9)	633 (44.9)
3–4	9 (2.3)	11 (1.1)	20 (1.4)
Lifetime History of Psychiatric Diagnoses n, (%)			
None	305 (76.6)	767 (75.9)	1072 (76.1)
Anxiety	27 (6.8)	78 (7.7)	105 (7.5)
Depression	5 (1.3)	13 (1.3)	18 (1.3)
Sleep disorders	8 (2.0)	10 (1.0)	18 (1.3)
ADHD	7 (1.8)	4 (0.4)	11 (0.8)
Other	46 (11.6)	139 (13.7)	185 (13.1)
Currently Psychiatric Medication (Yes %)	7.0	9.5	124 (8.8)
Currently Substance Use n (%)			
None	178 (44.7)	586 (58.0)	764 (54.2)
Alcohol	26 (6.5)	46 (4.5)	72 (5.1)
Tobacco	22 (5.5)	22 (2.2)	44 (3.1)
Caffeine	89 (22.4)	265 (26.2)	354 (25.1)
Vaping	2 (0.5)	3 (0.3)	5 (0.4)
Mix	81 (20.4)	89 (8.8)	170 (12.1)

Note: * As per Colombia’s official Statistics Department, DANE, socioeconomic status (SES) is determined based on various factors, including land use, access to public utilities, road accessibility, terrain, property value, and property features within the household’s living space. SES 1 represents the lowest socioeconomic status, while SES 6 signifies the highest.

**Table 2:** Prevalence and Mean Values of Social Jet Lag (SJL) and Mid-Sleep (MSFsc) across Chronotype Quintiles.

Quintile	Chronotype	SJL Prevalence (%)	Average SJL (Hours)	Average Midpoint of Sleep (MSFsc)
Q1	Extreme Morning	85.5%	5.26 h	3:00 A.M.
Q2	Morning	81.3%	5.10 h	4:00 A.M.
Q3	Neutral	87.3%	3.78 h	5:30 A.M.
Q4	Evening	84.6%	3.59 h	7:15 A.M.

Note: Values represent means within each chronotype quartile (Q1–Q4) based on MSFsc (midpoint of sleep on free days corrected for sleep debt), not overall sample averages. Q5 was excluded due to one atypical mid-sleep value.

**Table 3:** Differences in Work-Related Variables Affecting Sleep Patterns Between Genders.

	Men	Women	Total Sample	*p* Value
Rotating shifts in the last three months (Yes %)	27.1	26.8	379 (26.9)	>0.05 *
Usual Work Shift (%)				
Day Shift	57.0	46.7	699 (49.6)	
Night Shift	43.0	53.3	710 (50.4)	<0.01 *
Work in Non-workday (Yes %)	42.7	43.2	607 (43.1)	>0.05 *
Working hours during a usual week n, (%)				
Up to 36	235 (59.0)	660 (65.3)	895 (63.5)	
37 to 48	138 (34.7)	277 (27.4)	415 (29.5)	
More than 48	25 (6.3)	74 (7.3%)	99 (7.0)	<0.05 *
Sleep Duration workdays—Hours Mean (SD)	3.73 (3.99)	3.67 (3.52)	3.68 (3.65)	>0.05 **
Sleep Duration Non-workday—Hours Mean (SD)	7.43 (2.54)	7.70 (2.26)	7.63 (2.34)	>0.05 **
Number of Free Days During Week	3.2 (2.7)	3.7 (2.6)	3.64 (2.6)	<0.05 **

Note: * According to Chi-Square (χ2) test; ** According to Student *t*-test.

**Table 4:** Spearman’s Correlation among sleep variables and SJL.

	SJL	Sleep Duration Non-Workday (Hours)	Sleep Duration Workdays (Hours)	Age (Years)
Chronotype (MSFsc)	−0.311 ***	0.523 ***	0.839 ***	−0.189 **
Number of working days during a usual week	0.076 ***	−0.081 ***	−0.127 **	0.437 ***
Free Days	−0.076 ***	0.081 ***	0.127 ***	−0.437 ***
Social Jet Lag		0.583 **	−0.736 ***	−0.029 *
Sleep Duration Non-workday (Hours)			−0.023 *	−0.198 ***
Sleep Duration Workday (Hours)				−0.093 ***

Note: Positive correlations (r > 0) indicate that as one variable increases, the other also increases. Negative correlations (r < 0) indicate that as one variable increases, the other decreases. Correlations are significant at the levels of *p* < 0.05 (*), *p* < 0.01 (**), and *p* < 0.001 (***).

**Table 5:** Multiple Linear Regression Analysis of Variables Associated with Social Jet Lag.

Best Model—Outcome: SJL Lag * R^2^ = 0.158	β **	SE	*p* Value	CI (95%)
Variables–Risk Factors				
Chronotype (MSFsc)	−0.02	0.002	<0.001	−0.025 to −0.018
Age (years)	−3.28	0.84	<0.001	−4.942 to −1.618
Number of working days during a usual week	6.46	2.10	<0.01	2.340 to 10.583

Note: * SJL is expressed in minutes; ** β, unstandardized coefficients; MSFsc = indicator of chronotype, defined as the midpoint of sleep on free days adjusted for the sleep debt accrued during the workweek.

**Table 6:** One-way ANOVA results.

Dependent Variable	Independent Variable	Categories or Compared Factors	F	*p*
Social jetlag (minutes)	Beverage consumption	Coffee, tea, energy drinks, alcoholic beverages, soft drinks, juices	2.46	0.031
Social jetlag (minutes)	Psychoactive substance use	Tobacco, cannabis, anxiolytics, antidepressants, stimulants, others	2.46	0.031
Social jetlag (minutes)	Personal history of mental health disorders	Depression, anxiety, sleep disorders, chronic stress, other diagnoses, none	2.28	0.045

Note: The F-values and *p*-values reported correspond to the results of one-way ANOVA tests for differences in social jetlag (minutes) based on beverage consumption, psychoactive substance use, and personal history of mental health disorders. A post hoc Bonferroni test was conducted, but no significant differences were found between specific groups. Significant differences are marked with *p*-values less than 0.05.

## Data Availability

The data supporting the results of this study will be available upon request to the corresponding author. Due to privacy, legal, or ethical restrictions, the data are not publicly available, but the reason for these restrictions will be provided. Contact information will be provided so researchers can request access to the data.

## Supplementary Materials

The following supporting information can be downloaded at: https://www.mdpi.com/article/10.3390/clockssleep7040064/s1, Figure S1: Distribution of chronotypes based on mid-sleep time (MSFsc) across quintiles (Q1–Q5).

## References

[1] Golombek D.A., Rosenstein R.E. (2010). Physiology of circadian entrainment. Physiol. Rev..

[2] Grandin L.D., Alloy L.B., Abramson L.Y. (2006). The social zeitgeber theory, circadian rhythms, and mood disorders: Review and evaluation. Clin. Psychol. Rev..

[3] Beersma D.G., Gordijn M.C. (2007). Circadian control of the sleep-wake cycle. Physiol. Behav..

[4] Crowley S.J., Acebo C., Carskadon M.A. (2007). Sleep, circadian rhythms, and delayed phase in adolescence. Sleep Med..

[5] Roenneberg T., Pilz L.K., Zerbini G., Winnebeck E.C. (2019). Chronotype and social jetlag: A (self-) critical review. Biology.

[6] Otsuki R., Matsui K., Yoshiike T., Nagao K., Utsumi T., Tsuru A., Ayabe N., Hazumi M., Fukumizu M., Kuriyama K. (2022). Decrease in social zeitgebers is associated with worsened delayed sleep-wake phase disorder: Findings during the pandemic in Japan. Front. Psychiatry.

[7] Wright K.P., McHill A.W., Birks B.R., Griffin B.R., Rusterholz T., Chinoy E.D. (2013). Entrainment of the human circadian clock to the natural light-dark cycle. Curr. Biol..

[8] Rutters F., Lemmens S.G., Adam T.C., Bremmer M.A., Elders P.J., Nijpels G., Dekker J.M. (2014). Is social jetlag associated with an adverse endocrine, behavioral, and cardiovascular risk profile?. J. Biol. Rhythms.

[9] Caliandro R., Streng A.A., van Kerkhof L.W.M., van der Horst G.T.J., Chaves I. (2021). Social Jetlag and Related Risks for Human Health: A Timely Review. Nutrients.

[10] Montaruli A., Castelli L., Mulè A., Scurati R., Esposito F., Galasso L., Roveda E. (2021). Biological Rhythm and Chronotype: New Perspectives in Health. Biomolecules.

[11] Silva C.M., Mota M.C., Miranda M.T., Paim S.L., Waterhouse J., Crispim C.A. (2016). Chronotype, social jetlag and sleep debt are associated with dietary intake among Brazilian undergraduate students. Chronobiol. Int..

[12] Mota M.C., Silva C.M., Balieiro L.C.T., Fahmy W.M., Crispim C.A. (2017). Social jetlag and metabolic control in non-communicable chronic diseases: A study addressing different obesity statuses. Sci. Rep..

[13] Fárková E., Šmotek M., Bendová Z., Manková D., Kopřivová J. (2021). Chronotype and social jet-lag in relation to body weight, appetite, sleep quality and fatigue. Biol. Rhythm. Res..

[14] Wittmann M., Dinich J., Merrow M., Roenneberg T. (2006). Social jetlag: Misalignment of biological and social time. Chronobiol. Int..

[15] Knapen S.E., Riemersma-van der Lek R.F., Antypa N., Meesters Y., Penninx B.W.J.H., Schoevers R.A. (2018). Social jetlag and depression status: Results obtained from the Netherlands Study of Depression and Anxiety. Chronobiol. Int..

[16] Levandovski R., Dantas G., Fernandes L.C., Caumo W., Torres I., Roenneberg T., Hidalgo M.P.L., Allebrandt K.V. (2011). Depression scores associate with chronotype and social jetlag in a rural population. Chronobiol. Int..

[17] Tamura N., Okamura K. (2023). Social Jetlag as a Predictor of Depressive Symptoms among Japanese Adolescents: Evidence from the Adolescent Sleep Health Epidemiological Cohort. Sleep Health.

[18] Hasler B.P., McClung C.A. (2021). Delayed circadian rhythms and substance abuse: Dopamine transmission’s time has come. J. Clin. Investig..

[19] Hasler B.P., Smith L.J., Cousins J.C., Bootzin R.R. (2012). Circadian rhythms, sleep, and substance abuse. Sleep Med. Rev..

[20] Roenneberg T., Wirz-Justice A., Merrow M. (2003). Life between clocks: Daily temporal patterns of human chronotypes. J. Biol. Rhythms.

[21] Ghotbi N., Pilz L.K., Winnebeck E.C., Vetter C., Zerbini G., Lenssen D., Frighetto G., Salamanca M., Costa R., Montagnese S. (2020). The µMCTQ: An Ultra-Short Version of the Munich ChronoType Questionnaire. J. Biol. Rhythms.

[22] Chattu V., Manzar M., Kumary S., Burman D., Spence D., Pandi-Perumal S. (2019). The Global Problem of Insufficient Sleep and Its Serious Public Health Implications. Healthcare.

[23] Ghotbi N., Rabenstein A., Pilz L.K., Rüther T., Roenneberg T. (2023). Chronotype, Social Jetlag, and Nicotine Use. J. Biol. Rhythms.

[24] Carone C.M.M., Silva B.D.P., Rodrigues L.T., Tavares P.S., Carpena M.X., Santos I.S. (2020). Fatores associados a distúrbios do sono em estudantes universitários. Cad. Saúde Pública.

[25] Lopes X.F.M., Araújo M.F.S., Lira N.C.C., Dantas D.S., Souza J.C. (2022). Social, biological and behavioral factors associated with social jet lag and sleep duration in university students from a low urbanized city. J. Multidiscip. Healthc..

[26] Søvold L.E., Naslund J.A., Kousoulis A.A., Saxena S., Qoronfleh M.W., Grobler C., Münter L. (2021). Prioritizing the mental health and well-being of healthcare workers: An urgent global public health priority. Front. Public Health.

[27] Sato K., Kuroda S., Owan H. (2020). Mental health effects of long work hours, night and weekend work, and short rest periods. Soc. Sci. Med..

[28] Haraszti R.Á., Ella K., Gyöngyösi N., Roenneberg T., Káldi K. (2014). Social Jetlag Negatively Correlates with Academic Performance in Undergraduates. Chronobiol. Int..

[29] Roenneberg T., Allebrandt K.V., Merrow M., Vetter C. (2012). Social Jetlag and Obesity. Curr. Biol..

[30] Arber S., Bote M., Meadows R. (2009). Gender and socio-economic patterning of self-reported sleep problems in Britain. Soc. Sci. Med..

[31] Magnusdottir S., Magnusdottir I., Gunnlaugsdottir A.K., Hilmisson H., Hrolfsdottir L., Paed A.E.E.M. (2024). Sleep duration and social jetlag in healthy adolescents. Association with anxiety, depression, and chronotype: A pilot study. Sleep Breath..

[32] Giannotti F., Cortesi F., Sebastiani T., Ottaviano S. (2002). Circadian preference, sleep and daytime behaviour in adolescence. J. Sleep Res..

[33] Monk T.H., Buysse D.J., Potts J.M., DeGrazia J.M., Kupfer D.J. (2004). Morningness-eveningness and lifestyle regularity. Chronobiol. Int..

[34] Porcheret K., Wald L., Fritschi L., Gerkema M., Gordijn M., Merrow M., Rajaratnam S.M.W., Rock D., Sletten T.L., Warman G. (2018). Chronotype and environmental light exposure in a student population. Chronobiol. Int..

[35] Wang S., Wang H., Deng X., Lei X. (2023). Validation of the Munich Chronotype Questionnaire (MCTQ) in Chinese college freshmen based on questionnaires and actigraphy. Chronobiol. Int..

[36] Hsiao F.C., Wang Y.C., Reiter E., Wu C.W. (2025). The Linkage between Chronotype, Social Jetlag, and Responses to Sleep Inertia. Sci. Rep..

[37] Gottlieb E., Gahan L., Danoff-Burg S., Rus H., Watson N., Raymann R. (2022). Social Jetlag Decreases Across the Lifespan: A Longitudinal Big Data Analysis of Objective Sleep. Sleep.

[38] Casjens S., Brenscheidt F., Tisch A., Beermann B., Brüning T., Behrens T., Rabstein S. (2022). Social jetlag and sleep debts are altered in different rosters of night shift work. PLoS ONE.

[39] Juda M., Vetter C., Roenneberg T. (2013). The Munich chronotype questionnaire for shift-workers (MCTQShift). J. Biol. Rhythms.

[40] Diogo F.M.C., Bessa Z.C.M., Galina S.D., de Oliveira M.L.C., da Silva-Júnior E.L.R., Valdez P., de Azevedo C.V.M. (2024). Sex Differences in Temporal Sleep Patterns, Social Jetlag, and Attention in High School Adolescents. Sleep Sci..

[41] Kim H.-Y. (2013). Statistical notes for clinical researchers: Assessing normal distribution (2) using skewness and kurtosis. Restor. Dent. Endod..

[42] Camargo A., Olmos J., Higuera Dagovett E., Vargas R., Barreto R. (2019). Papel de los profesionales de la salud en el diseño, obtención y entendimiento del consentimiento informado: Una revisión. Rev. UDCA Actual. Divulg. Científica.

