# Supplementary material for: Sleep and Psychosocial Risk Factors Associated with Social Jet Lag and Sleep Duration Among Colombian University Students
Source: Clocks Sleep. 2025 Nov 7;7(4):64. doi: 10.3390/clockssleep7040064 (PMC12641817; doi:10.3390/clockssleep7040064)
Supplement: Supplementary file 1 [file clockssleep-07-00064-s001.zip › clockssleep-3881742-supplementary.pdf]

Figure S1. Average mid-sleep time (MSFsc, clock time) across chronotype quintiles (Q1–Q5)

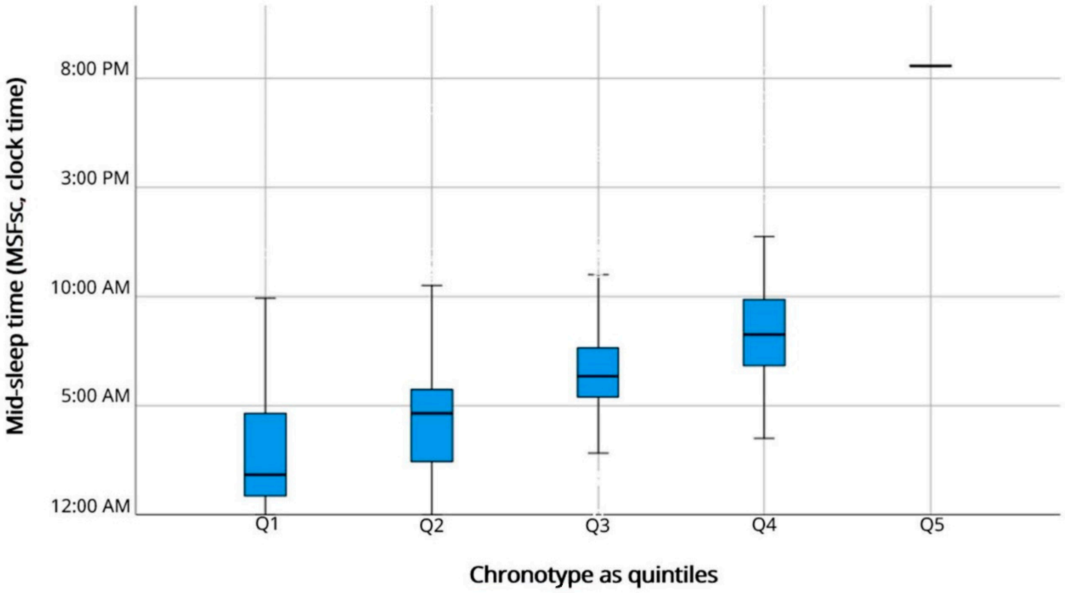

Distribution of Chronotypes Based on Mid-Sleep Time (MSFsc) across Quintiles (Q1–Q5).

Note* Note: Chronotype (MSFsc) was categorized into five quintiles: Q1 (early chronotype, lowest MSFsc), Q2 (early intermediate), Q3 (late intermediate), Q4 (late), and Q5 (very late). Q5 included only one participant with a potentially atypical mid-sleep value, which may limit interpretation for this group.
